# Supplementary material for: A Likelihood Approach for Real-Time Calibration of Stochastic Compartmental Epidemic Models
Source: PLoS Comput Biol. 2017 Jan 17;13(1):e1005257. doi: 10.1371/journal.pcbi.1005257 (PMC5240920; doi:10.1371/journal.pcbi.1005257)
Supplement: S1 File — (TAR.GZ) [file pcbi.1005257.s014.tar.gz › HSPH_Online-SI-Revision/MSS10/n10-severe/n10-severe_NEW2_table-4.pdf]

1	newly 3 weeks spec	606.	556.45	{326., 674.}	14.9488	0.0801348	newly 3 weeks cumul	1634.	1557.48	{1102., 1778.}	11.7699	0.0597965	newly 5 weeks spec	589.	501.55	{210., 648.}	21.1834	0.142561	newly 5 weeks cumul	2883.	2602.06	{1539., 3067.}	14.0423	0.0776969	newly 55 weeks cumul	5530.	4758.05	{2240., 5882.}	19.3553	0.122902
2	newly 3 weeks spec	693.	478.72	{344., 591.}	30.9206	0.179056	newly 3 weeks cumul	1675.	1296.12	{977., 1562.}	22.8967	0.127385	newly 5 weeks spec	711.	446.85	{283., 630.}	37.18	0.226735	newly 5 weeks cumul	3118.	2216.94	{1644., 2775.}	28.8987	0.162722	newly 57 weeks cumul	5958.	4268.72	{2589., 7216.}	37.4874	0.222291
3	newly 3 weeks spec	672.	494.9	{345., 644.}	27.506	0.153873	newly 3 weeks cumul	1714.	1445.54	{1049., 1821.}	19.4422	0.102735	newly 5 weeks spec	640.	361.16	{230., 509.}	44.125	0.269325	newly 5 weeks cumul	3081.	2249.99	{1680., 2765.}	27.6141	0.149301	newly 59 weeks cumul	4699.	3166.54	{2414., 4272.}	33.768	0.186032
4	newly 3 weeks spec	729.	468.34	{304., 610.}	35.9863	0.210098	newly 3 weeks cumul	1838.	1368.19	{859., 1838.}	27.7187	0.156612	newly 5 weeks spec	670.	325.54	{224., 438.}	51.4119	0.32724	newly 5 weeks cumul	3254.	2107.55	{1506., 2638.}	35.232	0.201145	newly 54 weeks cumul	5574.	2843.15	{2378., 3272.}	48.9926	0.296167
5	newly 3 weeks spec	577.	464.65	{343., 570.}	20.8683	0.109214	newly 3 weeks cumul	1549.	1458.62	{1034., 1814.}	15.9561	0.0756148	newly 5 weeks spec	572.	281.69	{168., 389.}	50.7535	0.328864	newly 5 weeks cumul	2731.	2119.22	{1652., 2567.}	22.5236	0.118012	newly 54 weeks cumul	5177.	2744.49	{2145., 3347.}	46.9869	0.282578
6	newly 3 weeks spec	667.	387.62	{144., 584.}	42.1259	0.305753	newly 3 weeks cumul	1499.	1088.78	{377., 1645.}	34.7885	0.23596	newly 5 weeks spec	615.	305.46	{100., 436.}	50.9073	0.375976	newly 5 weeks cumul	2818.	1751.1	{771., 2620.}	38.3172	0.26398	newly 55 weeks cumul	4913.	2694.63	{1781., 3239.}	46.5954	0.295918
7	newly 3 weeks spec	404.	289.62	{165., 415.}	30.9851	0.185303	newly 3 weeks cumul	1214.	1019.43	{602., 1377.}	23.7554	0.130034	newly 5 weeks spec	360.	173.08	{82., 260.}	51.9222	0.360646	newly 5 weeks cumul	2017.	1423.16	{903., 1898.}	30.3679	0.177263	newly 54 weeks cumul	3462.	1844.2	{1118., 2399.}	46.7302	0.292199
8	newly 3 weeks spec	687.	746.04	{652., 823.}	11.9971	0.0498128	newly 3 weeks cumul	1740.	1891.39	{1688., 2080.}	10.8328	0.0447295	newly 5 weeks spec	686.	741.95	{564., 841.}	15.2085	0.0646409	newly 5 weeks cumul	3148.	3409.29	{3040., 3685.}	11.3218	0.0474465	newly 58 weeks cumul	4943.	6255.68	{4765., 6890.}	29.7139	0.112752
9	newly 3 weeks spec	655.	464.12	{195., 637.}	35.8565	0.284638	newly 3 weeks cumul	1488.	1010.38	{476., 1343.}	39.2446	0.271224	newly 5 weeks spec	787.	598.63	{265., 865.}	30.6264	0.310726	newly 5 weeks cumul	3066.	2161.1	{1037., 2895.}	32.2551	0.242753	newly 62 weeks cumul	5852.	5339.41	{2842., 8631.}	43.4612	0.282633
10	newly 3 weeks spec	791.	952.25	{564., 1268.}	33.8344	0.131751	newly 3 weeks cumul	2024.	2464.19	{1612., 3069.}	31.1388	0.122607	newly 5 weeks spec	740.	690.36	{263., 1091.}	37.1865	0.191221	newly 5 weeks cumul	3581.	4023.9	{2647., 5370.}	27.3795	0.112295	newly 62 weeks cumul	5507.	5582.7	{3038., 8524.}	33.7654	0.149131
11	newly 3 weeks spec	764.	695.5	{258., 1216.}	40.3272	0.28712	newly 3 weeks cumul	1667.	1835.43	{430., 3477.}	49.1596	0.264134	newly 5 weeks spec	893.	360.8	{189., 529.}	60.2262	0.505156	newly 5 weeks cumul	3478.	2740.34	{1253., 4441.}	33.6947	0.238312	newly 61 weeks cumul	4839.	3390.85	{2593., 4579.}	30.018	0.215512
12	newly 3 weeks spec	676.	597.9	{400., 833.}	24.1479	0.118661	newly 3 weeks cumul	1411.	1332.62	{1016., 1791.}	22.533	0.103886	newly 5 weeks spec	750.	671.83	{390., 909.}	24.356	0.127949	newly 5 weeks cumul	2924.	2684.98	{1908., 3498.}	18.0109	0.0876288	newly 61 weeks cumul	5377.	5467.25	{3063., 8134.}	36.1973	0.161224
13	newly 3 weeks spec	785.	838.74	{506., 1103.}	23.5618	0.100988	newly 3 weeks cumul	1843.	2035.93	{1332., 2673.}	24.2339	0.100043	newly 5 weeks spec	871.	638.22	{379., 797.}	28.597	0.161404	newly 5 weeks cumul	3619.	3469.86	{2403., 4256.}	16.2006	0.0771537	newly 58 weeks cumul	6232.	4748.4	{3188., 5305.}	27.8947	0.14549
14	newly 3 weeks spec	790.	1021.57	{857., 1142.}	30.8165	0.117378	newly 3 weeks cumul	2006.	2612.7	{2234., 3112.}	31.9232	0.120039	newly 5 weeks spec	648.	700.22	{507., 1052.}	25.0247	0.0998737	newly 5 weeks cumul	3522.	4227.24	{3614., 4755.}	21.4594	0.0851439	newly 60 weeks cumul	4964.	5742.2	{4633., 8313.}	19.8348	0.0705759
15	newly 3 weeks spec	973.	460.56	{86., 759.}	52.7215	0.499756	newly 3 weeks cumul	2131.	1149.13	{303., 1906.}	46.7804	0.389096	newly 5 weeks spec	967.	392.87	{113., 572.}	59.3723	0.54337	newly 5 weeks cumul	4148.	1995.55	{575., 2997.}	51.8913	0.426695	newly 64 weeks cumul	6236.	3095.2	{2307., 3675.}	50.3656	0.381376
16	newly 3 weeks spec	860.	741.85	{342., 1040.}	31.457	0.178702	newly 3 weeks cumul	2325.	2193.66	{1244., 2730.}	22.0972	0.115063	newly 5 weeks spec	767.	552.36	{132., 905.}	42.6128	0.332169	newly 5 weeks cumul	3984.	3407.78	{1695., 4613.}	29.2008	0.16491	newly 60 weeks cumul	5671.	5215.29	{2075., 7752.}	45.7085	0.248811
17	newly 3 weeks spec	584.	445.77	{289., 589.}	25.8202	0.142964	newly 3 weeks cumul	1339.	1186.69	{740., 1607.}	21.0792	0.10815	newly 5 weeks spec	581.	404.67	{283., 541.}	30.9862	0.17323	newly 5 weeks cumul	2523.	2034.21	{1372., 2598.}	21.2243	0.114062	newly 60 weeks cumul	5901.	3353.99	{2562., 4377.}	43.1623	0.253038
18	newly 3 weeks spec	574.	435.29	{296., 559.}	25.6185	0.143552	newly 3 weeks cumul	1527.	1208.32	{804., 1501.}	23.277	0.128054	newly 5 weeks spec	639.	333.92	{226., 439.}	47.7433	0.303394	newly 5 weeks cumul	2806.	1943.03	{1380., 2414.}	30.9041	0.174218	newly 58 weeks cumul	5958.	2819.22	{2189., 3435.}	52.6818	0.336789
19	newly 3 weeks spec	718.	489.33	{389., 598.}	31.8482	0.172518	newly 3 weeks cumul	1880.	1519.79	{1220., 1798.}	19.4144	0.0983645	newly 5 weeks spec	617.	301.59	{235., 375.}	51.1199	0.318249	newly 5 weeks cumul	3238.	2220.88	{1772., 2617.}	31.412	0.167667	newly 54 weeks cumul	5565.	2895.98	{2517., 3228.}	47.9608	0.285921
20	newly 3 weeks spec	409.	365.51	{159., 529.}	28.3692	0.165446	newly 3 weeks cumul	1135.	1025.22	{476., 1460.}	28.7154	0.158414	newly 5 weeks spec	432.	291.8	{134., 395.}	32.875	0.230618	newly 5 weeks cumul	2027.	1656.89	{775., 2296.}	25.6265	0.155146	newly 54 weeks cumul	5250.	2505.3	{1691., 3159.}	52.28	0.347382
21	newly 3 weeks spec	797.	827.14	{490., 1092.}	24.0803	0.109295	newly 3 weeks cumul	1935.	2084.44	{1289., 2702.}	24.7039	0.108556	newly 5 weeks spec	789.	599.44	{327., 818.}	30.5906	0.173184	newly 5 weeks cumul	3576.	3439.	{2361., 4519.}	18.6834	0.0902497	newly 62 weeks cumul	5739.	4635.93	{2963., 5336.}	27.3032	0.140144
22	newly 3 weeks spec	515.	424.33	{99., 604.}	35.365	0.307299	newly 3 weeks cumul	1274.	1164.64	{432., 1615.}	34.4176	0.199948	newly 5 weeks spec	507.	388.07	{32., 656.}	46.1479	0.497462	newly 5 weeks cumul	2335.	1962.41	{542., 2607.}	31.0899	0.225318	newly 60 weeks cumul	4471.	4162.94	{677., 7155.}	54.5386	0.361475
23	newly 3 weeks spec	509.	429.35	{241., 604.}	24.8153	0.135244	newly 3 weeks cumul	1486.	1248.05	{680., 1685.}	24.7678	0.136521	newly 5 weeks spec	479.	315.31	{219., 430.}	34.6576	0.200126	newly 5 weeks cumul	2515.	1942.95	{1153., 2563.}	24.9777	0.139501	newly 59 weeks cumul	4951.	2782.68	{2119., 3380.}	43.7956	0.258284
24	newly 3 weeks spec	864.	678.	{237., 1047.}	32.0787	0.207146	newly 3 weeks cumul	1807.	1545.9	{507., 2310.}	30.207	0.186927	newly 5 weeks spec	1016.	617.32	{271., 887.}	39.5689	0.267935	newly 5 weeks cumul	3920.	2871.01	{1183., 4117.}	29.4487	0.192214	newly 60 weeks cumul	6534.	4277.8	{2982., 5440.}	34.5369	0.209873
25	newly 3 weeks spec	883.	554.16	{100., 1464.}	62.3737	0.512515	newly 3 weeks cumul	1516.	1054.52	{221., 2537.}	59.0343	0.417247	newly 5 weeks spec	1165.	640.21	{162., 1173.}	48.6841	0.42169	newly 5 weeks cumul	3941.	2365.32	{580., 4732.}	50.8389	0.406921	newly 63 weeks cumul	5681.	4240.11	{3083., 6161.}	29.6601	0.211074
26	newly 3 weeks spec	596.	994.04	{605., 1215.}	69.1846	0.218993	newly 3 weeks cumul	1544.	2329.31	{1567., 2752.}	53.8854	0.183096	newly 5 weeks spec	608.	910.16	{394., 1155.}	62.6941	0.216875	newly 5 weeks cumul	2767.	4269.98	{2695., 5107.}	57.5201	0.190577	newly 62 weeks cumul	4667.	6901.35	{3250., 8590.}	57.8202	0.195039
27	newly 3 weeks spec	646.	1182.96	{509., 1599.}	92.1362	0.282544	newly 3 weeks cumul	1397.	2843.75	{1166., 4195.}	113.213	0.317345	newly 5 weeks spec	776.	464.14	{147., 952.}	50.3608	0.355865	newly 5 weeks cumul	2980.	4132.54	{2405., 4904.}	46.4919	0.17056	newly 63 weeks cumul	6059.	4864.72	{3500., 5166.}	25.8287	0.128337
28	newly 3 weeks spec	775.	993.59	{873., 1111.}	28.2671	0.106438	newly 3 weeks cumul	1597.	1975.58	{1735., 2199.}	23.7708	0.0910353	newly 5 weeks spec	804.	1269.03	{1200., 1330.}	57.8396	0.197911	newly 5 weeks cumul	3259.	4491.84	{4171., 4821.}	37.8288	0.138689	newly 62 weeks cumul	5190.	8614.5	{8235., 9067.}	65.9827	0.219849
29	newly 3 weeks spec	901.	976.46	{422., 1644.}	37.1853	0.162067	newly 3 weeks cumul	1967.	2031.97	{912., 3258.}	33.666	0.156633	newly 5 weeks spec	1020.	889.12	{445., 1340.}	27.9294	0.149327	newly 5 weeks cumul	4131.	3967.57	{2237., 6242.}	27.9325	0.1334	newly 64 weeks cumul	5712.	5647.14	{3503., 8767.}	24.3498	0.109527
30	newly 3 weeks spec	883.	1288.34	{979., 1530.}	47.4315	0.164763	newly 3 weeks cumul	1653.	2291.04	{1737., 2737.}	40.7743	0.144857	newly 5 weeks spec	1189.	1539.68	{1452., 1645.}	29.7157	0.112204	newly 5 weeks cumul	4088.	5472.37	{4706., 6065.}	34.8041	0.128441	newly 64 weeks cumul	6316.	8825.65	{8318., 9175.}	39.9257	0.1452
31	newly 3 weeks spec	575.	527.15	{312., 785.}	25.0835	0.131897	newly 3 weeks cumul	1530.	1554.56	{941., 2035.}	25.2209	0.125419	newly 5 weeks spec	506.	361.88	{161., 842.}	50.9486	0.294398	newly 5 weeks cumul	2641.	2368.82	{1467., 3639.}	25.1609	0.129777	newly 61 weeks cumul	4464.	3490.59	{1936., 7912.}	48.179	0.247588
32	newly 3 weeks spec	390.	315.96	{202., 412.}	22.5744	0.128359	newly 3 weeks cumul	1183.	1071.82	{753., 1354.}	16.3466	0.0873737	newly 5 weeks spec	341.	196.4	{122., 282.}	42.4047	0.271978	newly 5 weeks cumul	1958.	1527.55	{1033., 1915.}	22.7768	0.127671	newly 54 weeks cumul	3806.	1979.56	{1466., 2482.}	47.9884	0.299258
33	newly 3 weeks spec	499.	416.49	{270., 556.}	21.1403	0.109329	newly 3 weeks cumul	1415.	1246.26	{890., 1601.}	17.9661	0.0899334	newly 5 weeks spec	521.	291.99	{187., 404.}	43.9559	0.26859	newly 5 weeks cumul	2457.	1893.06	{1418., 2400.}	23.7094	0.125565	newly 54 weeks cumul	5031.	2684.49	{2015., 3573.}	46.641	0.282931
34	newly 3 weeks spec	526.	443.9	{380., 506.}	15.7681	0.0770323	newly 3 weeks cumul	1521.	1338.94	{1168., 1483.}	12.2446	0.0581858	newly 5 weeks spec	469.	359.5	{311., 412.}	23.3475	0.11784	newly 5 weeks cumul	2539.	2100.71	{1878., 2312.}	17.2623	0.0837173	newly 54 weeks cumul	4810.	3329.32	{3059., 3554.}	30.7834	0.160642
35	newly 3 weeks spec	688.	819.82	{679., 927.}	21.0465	0.0817357	newly 3 weeks cumul	1586.	1814.46	{1533., 2024.}	16.8008	0.0669628	newly 5 weeks spec	769.	991.76	{881., 1082.}	30.1717	0.113991	newly 5 weeks cumul	3166.	3764.05	{3261., 4133.}	20.0534	0.0786848	newly 61 weeks cumul	5296.	8423.72	{8310., 8801.}	60.1178	0.204045
36	newly 3 weeks spec	502.	427.26	{269., 562.}	22.0598	0.115687	newly 3 weeks cumul	1350.	1229.16	{763., 1591.}	19.7422	0.0992544	newly 5 weeks spec	557.	315.78	{192., 413.}	43.307	0.267455	newly 5 weeks cumul	2471.	1925.22	{1354., 2396.}	23.5055	0.127006	newly 57 weeks cumul	5430.	2796.3	{2082., 3261.}	48.5028	0.294821
37	newly 3 weeks spec	552.	651.46	{262., 945.}	42.0942	0.191236	newly 3 weeks cumul	1345.	1677.49	{860., 2273.}	44.1896	0.192207	newly 5 weeks spec	542.	586.24	{215., 996.}	57.2952	0.286316	newly 5 weeks cumul	2492.	2907.81	{1536., 4268.}	40.7508	0.181495	newly 60 weeks cumul	4762.	4958.05	{2482., 8012.}	50.9448	0.235416
38	newly 3 weeks spec	663.	447.58	{269., 600.}	32.5973	0.195894	newly 3 weeks cumul	1606.	1184.24	{671., 1576.}	27.599	0.161318	newly 5 weeks spec	672.	398.97	{251., 532.}	42.4211	0.258357	newly 5 weeks cumul	3051.	2021.89	{1237., 2690.}	33.7388	0.19728	newly 60 weeks cumul	6153.	3362.06	{2360., 3791.}	48.667	0.294957
39	newly 3 weeks spec	517.	453.02	{389., 526.}	13.9304	0.0677248	newly 3 weeks cumul	1361.	1286.13	{1096., 1444.}	9.71712	0.04535	newly 5 weeks spec	517.	400.77	{323., 466.}	22.6132	0.116344	newly 5 weeks cumul	2422.	2120.57	{1834., 2362.}	13.2605	0.0642539	newly 56 weeks cumul	5258.	3726.95	{3346., 4058.}	29.1185	0.151403
40	newly 3 weeks spec	579.	412.28	{285., 538.}	29.0432	0.16303	newly 3 weeks cumul	1495.	1220.83	{869., 1600.}	21.7505	0.116898	newly 5 weeks spec	560.	286.14	{181., 381.}	48.9036	0.310727	newly 5 weeks cumul	2700.	1863.81	{1311., 2370.}	30.97	0.171907	newly 56 weeks cumul	5592.	2576.61	{2042., 3137.}	53.9233	0.34392
41	newly 3 weeks spec	748.	1028.46	{745., 1266.}	40.4439	0.143838	newly 3 weeks cumul	1534.	2017.25	{1445., 2512.}	35.3214	0.12838	newly 5 weeks spec	1008.	1141.56	{853., 1346.}	20.2044	0.0836	newly 5 weeks cumul	3552.	4391.84	{3416., 5092.}	26.7838	0.102471	newly 64 weeks cumul	5882.	6837.01	{5415., 8801.}	21.5775	0.0841515
42	newly 3 weeks spec	764.	455.27	{176., 700.}	41.0196	0.283002	newly 3 weeks cumul	1835.	1252.71	{417., 1991.}	36.9891	0.245111	newly 5 weeks spec	835.	334.57	{146., 490.}	59.9317	0.438706	newly 5 weeks cumul	3509.	2003.05	{1007., 2900.}	42.9168	0.278218	newly 55 weeks cumul	5877.	2785.78	{2161., 3326.}	52.5986	0.339092
43	newly 3 weeks spec	917.	783.94	{376., 1245.}	32.6957	0.181895	newly 3 weeks cumul	2107.	1815.62	{847., 2695.}	28.8372	0.163097	newly 5 weeks spec	846.	681.	{321., 1220.}	38.2057	0.213189	newly 5 weeks cumul	3927.	3294.81	{1736., 5209.}	31.0102	0.168775	newly 63 weeks cumul	5868.	4885.89	{2818., 8931.}	37.3874	0.190814
44	newly 3 weeks spec	531.	533.37	{403., 655.}	16.2919	0.0743008	newly 3 weeks cumul	1403.	1424.57	{1108., 1711.}	14.3542	0.0633508	newly 5 weeks spec	507.	498.57	{305., 625.}	17.7495	0.0857908	newly 5 weeks cumul	2442.	2458.5	{1817., 2902.}	14.8575	0.0665876	newly 57 weeks cumul	4771.	4118.11	{2648., 4733.}	17.1614	0.088941
45	newly 3 weeks spec	842.	955.91	{782., 1086.}	20.7423	0.0877863	newly 3 weeks cumul	1994.	2319.8	{1967., 2570.}	20.1605	0.0807083	newly 5 weeks spec	777.	914.83	{552., 1064.}	29.5277	0.129544	newly 5 weeks cumul	3630.	4229.54	{3597., 4745.}	22.4942	0.0922421	newly 60 weeks cumul	5942.	7265.28	{4629., 8291.}	31.5436	0.12775
46	newly 3 weeks spec	429.	398.38	{248., 555.}	22.8345	0.14602	newly 3 weeks cumul	1207.	1141.12	{691., 1570.}	23.6669	0.131647	newly 5 weeks spec	442.	310.06	{175., 413.}	30.7104	0.210275	newly 5 weeks cumul	2135.	1814.82	{1063., 2399.}	20.7288	0.128761	newly 59 weeks cumul	4374.	2682.98	{2038., 3266.}	38.6607	0.239683
47	newly 3 weeks spec	432.	449.65	{313., 590.}	21.3542	0.0951819	newly 3 weeks cumul	1154.	1289.39	{866., 1700.}	23.3492	0.0964858	newly 5 weeks spec	487.	335.9	{238., 438.}	31.1992	0.174961	newly 5 weeks cumul	2160.	2032.1	{1520., 2537.}	15.5657	0.0761136	newly 60 weeks cumul	4498.	2869.78	{2323., 3345.}	36.1988	0.199604
48	newly 3 weeks spec	691.	646.21	{448., 789.}	15.11	0.074512	newly 3 weeks cumul	1624.	1533.88	{1173., 1836.}	12.1946	0.0582477	newly 5 weeks spec	702.	741.	{387., 956.}	31.0313	0.14407	newly 5 weeks cumul	3044.	2997.7	{2068., 3703.}	16.9336	0.0793155	newly 57 weeks cumul	6315.	6414.05	{3047., 8393.}	31.5587	0.152753
49	newly 3 weeks spec	507.	540.7	{310., 738.}	28.4181	0.128339	newly 3 weeks cumul	1374.	1510.99	{837., 2051.}	28.8144	0.125907	newly 5 weeks spec	548.	389.99	{198., 766.}	44.823	0.256369	newly 5 weeks cumul	2534.	2379.39	{1378., 3271.}	21.6689	0.109118	newly 59 weeks cumul	4591.	3626.28	{2288., 7147.}	42.3803	0.216365
50	newly 3 weeks spec	1068.	966.96	{554., 1256.}	20.3446	0.105765	newly 3 weeks cumul	2428.	2407.35	{1497., 3208.}	21.465	0.100449	newly 5 weeks spec	913.	642.98	{428., 813.}	29.7262	0.168786	newly 5 weeks cumul	4440.	3910.07	{2564., 4760.}	16.5953	0.0864256	newly 64 weeks cumul	6234.	4883.47	{3175., 5577.}	22.1577	0.11742
